# Serum neudesin is elevated in pregnancies complicated by pregestational type 2 diabetes: an exploratory cross-sectional controlled study: Neudesin in pregestational diabetes during pregnancy

**DOI:** 10.1186/s12884-026-09743-5

**Published:** 2026-07-31

**Authors:** Zuhal Köksal, Funda Güçel, Şeyma Sarışen

**Affiliations:** 1 Ankara Etlik City Hospital Internal Medicine, Ankara, Turkey; 2 Ankara Etlik City Hospital Medical Biochemistry, Ankara, Turkey

**Keywords:** Gestational diabetes mellitus, Pregestational type 2 diabetes mellitus, Neudesin, Adipokines, Pregnancy, Metabolic bio marker

## Abstract

**Objective:**

Neudesin has been linked to adipose tissue activity, glucose metabolism, and insulin resistance. Evidence on its behavior in diabetic pregnancies is limited, particularly in women with pregestational type 2 diabetes mellitus (PREGDM). This exploratory cross-sectional controlled study evaluated serum neudesin concentrations in pregnancies complicated by pregestational type 2 diabetes mellitus (PREGDM) or gestational diabetes mellitus (GDM), and in non-diabetic pregnant controls without major systemic disease, while examining the influence of gestational timing.

**Methods:**

This study included 90 pregnant women: 30 with PREGDM, 30 with GDM, and 30 non-diabetic controls without major systemic disease. Controls were recruited in two planned gestational-age strata, including early pregnancy and 24–28 weeks, to provide clinically relevant timing-aware comparisons. Serum neudesin concentrations were measured using a human neudesin enzyme-linked immunosorbent assay kit (BT LAB, catalogue no. E4258Hu) and reported as ng/mL. Overall group comparisons were supplemented by gestational-timing-aware analyses and exploratory multivariable log-neudesin regression.

**Results:**

Overall median serum neudesin was highest in the PREGDM group (5.67 [4.72–6.21] ng/mL), followed by the GDM group (2.80 [2.70–3.10] ng/mL) and controls (2.17 [1.77–2.89] ng/mL; *p* < 0.001). In gestational-timing-aware comparisons, PREGDM remained higher than early-pregnancy controls (5.67 [4.72–6.21] vs. 1.70 [1.37–2.15] ng/mL; Hodges–Lehmann difference, 3.70 ng/mL; 95% CI, 3.15–4.35; *p* < 0.001). In contrast, GDM did not differ from controls sampled at 24–28 weeks (2.80 [2.70–3.10] vs. 2.85 [2.28–3.03] ng/mL; Hodges–Lehmann difference, 0.04 ng/mL; 95% CI, − 0.20 to 0.40; *p* = 0.791). In the diabetic-only model adjusted for gestational age and age, the PREGDM-GDM contrast was attenuated (GMR, 1.31; 95% CI, 0.61–2.85; *p* = 0.490). In the all-participant adjusted model, PREGDM remained associated with higher neudesin than controls (GMR, 3.11; 95% CI, 2.57–3.76; *p* < 0.001), whereas the GDM-control contrast was not significant (GMR, 1.12; 95% CI, 0.96–1.30; *p* = 0.149).

**Conclusion:**

Serum neudesin was most consistently elevated in pregnancies complicated by PREGDM. The apparent overall GDM-control difference was not retained after timing-aware comparison or adjustment. Given the modest sample size, neudesin should be considered an exploratory complementary metabolic marker rather than a standalone diagnostic biomarker.

## Introduction

Diabetes mellitus in pregnancy is one of the major metabolic disorders with important effects on maternal, fetal, and neonatal outcomes. Hyperglycemia detected during pregnancy may present either as gestational diabetes mellitus (GDM), first recognized during pregnancy, or as pregestational diabetes mellitus, which is already present before conception. In both conditions, the underlying pathophysiology is closely related to insulin resistance and impaired glucose metabolism; however, pregestational diabetes carries a distinct clinical burden because chronic metabolic exposure begins early in pregnancy [1].

As pregnancy advances, physiological insulin resistance develops as an adaptive mechanism that helps ensure adequate transfer of glucose and nutrients to the fetus. When pancreatic beta-cell compensation is insufficient, maternal hyperglycemia may occur and GDM may develop. GDM is associated with short- and long-term complications, including macrosomia, preeclampsia, cesarean delivery, neonatal hypoglycemia, and an increased risk of type 2 diabetes later in the mother’s life [1, 2]. For this reason, early recognition of glycemic disturbances and a better understanding of metabolic risk markers are clinically relevant.

Adipose tissue is not merely a passive site of energy storage; it is also an active endocrine organ involved in metabolic and inflammatory regulation. Adipokines released from adipose tissue influence appetite regulation, energy balance, insulin sensitivity, inflammation, endothelial function, and glucose metabolism. Several reviews and clinical studies have therefore evaluated adipokines as diagnostic or risk-related markers in GDM and related pregnancy-metabolic states [3–8]. Evaluating newer adipokines together with conventional metabolic parameters may provide a more detailed view of metabolic alterations in diabetic pregnancies.

Neudesin, also known as neuron-derived neurotrophic factor, was initially described as a protein associated with the central nervous system. It has since been recognized as a secreted protein that may be linked to energy metabolism, adipose tissue function, obesity, and insulin resistance. Experimental and clinical studies suggest that neudesin may have a role in energy homeostasis, adipogenesis, glucose metabolism, and insulin sensitivity [9–12]. Increased serum neudesin levels have been reported in patients with type 2 diabetes, and this increase may be related to obesity and insulin resistance [12]. At the same time, studies in different age groups and metabolic settings have shown that neudesin levels may vary according to the metabolic context [10, 11].

Data on neudesin in GDM remain limited. Recent studies have suggested that maternal serum neudesin levels may be higher in pregnant women with GDM than in healthy pregnant controls and that neudesin may be associated with fasting glucose, HbA1c, insulin resistance indices, and body mass index [13, 14]. A more recent study evaluating both maternal and cord blood neudesin levels also reported higher maternal and fetal neudesin concentrations in pregnancies complicated by GDM than in controls [15]. These findings raise the possibility that neudesin may be a novel biomarker related to metabolic adaptation and insulin resistance in diabetic pregnancies.

GDM and pregestational diabetes differ in the timing and duration of hyperglycemia, baseline metabolic burden, and expected effects on pregnancy [16–18]. In pregestational diabetes, metabolic exposure begins before conception and continues through early organogenesis, whereas GDM is usually recognized later in pregnancy. Because PREGDM and GDM differ not only in glycemic status but also in the timing and cumulative duration of metabolic exposure, they provide a clinically informative setting in which to explore whether circulating neudesin is associated with diabetic pregnancy phenotypes. This exploratory study therefore aimed to compare serum neudesin levels among women with GDM, women with pregestational type 2 diabetes, and non-diabetic pregnant controls, and to evaluate the relationship between neudesin and selected maternal metabolic parameters.

## Materials and methods

This exploratory cross-sectional controlled clinical study evaluated serum neudesin levels in pregnant women with GDM, pregnant women with pregestational type 2 diabetes, and non-diabetic pregnant controls without major systemic disease. The study was conducted at the Internal Medicine Clinic of Ankara Etlik City Hospital. Pregnant women attending the Internal Medicine outpatient clinic were included and classified into three groups: GDM, PREGDM, and non-diabetic pregnancy.

The study included 90 pregnant women, with 30 participants in each group. The GDM group consisted of women older than 18 years who were diagnosed after an oral glucose tolerance test performed during pregnancy. The PREGDM group included pregnant women with a diagnosis of type 2 diabetes mellitus before pregnancy. The control group consisted of non-diabetic pregnant women with normal glucose tolerance during routine pregnancy follow-up. Because PREGDM was evaluated predominantly in early pregnancy whereas GDM was sampled at the time of diagnosis, usually at 24–28 weeks, controls were intentionally recruited in two prespecified gestational-age strata to provide clinically appropriate comparisons: 15 controls at 6–12 weeks and 15 controls at 24–28 weeks. Early-pregnancy controls were recruited from women without known gestational diabetes risk factors who attended the Internal Medicine outpatient clinic because of non-specific complaints, most commonly mild thyroid hormone abnormalities or dyspeptic symptoms. Women with known diabetes, major systemic disease, or conditions considered likely to substantially affect metabolic status were not included. For controls sampled at 6–12 weeks, serum was collected in early pregnancy and glucose tolerance status was subsequently verified by routine OGTT at 24–28 weeks. Only women with a normal follow-up OGTT and no diagnosis of GDM were retained in the control cohort; women with an abnormal OGTT or subsequent GDM were excluded. Thus, the overall control group spanned two planned sampling windows rather than representing an incidental gestational-age mismatch. All participants agreed to participate and provided written informed consent.

To minimize treatment-related confounding, eligibility for the PREGDM group was restricted to women with pregestational type 2 diabetes who had been managed with dietary measures alone and had not used insulin, metformin, or any other glucose-lowering medication before enrollment. Women with current or previous pharmacological glucose-lowering treatment were not included in the PREGDM group. Serum samples were obtained before initiation of any pregnancy-related glucose-lowering treatment. Likewise, GDM samples were collected at the time of diagnosis and before pharmacological treatment was started. Accordingly, at blood sampling, diet-only management was recorded in all PREGDM and GDM participants, and there was no exposure to oral antidiabetic agents, insulin, or combination therapy in either diabetic group.

Known duration of type 2 diabetes was recorded for all PREGDM participants and is reported in Table 1. Before enrolment, fundoscopic examination, microalbuminuria assessment, and routine laboratory testing were performed. Participants with diabetic retinopathy, microalbuminuria, clinically relevant renal dysfunction, or abnormal routine laboratory findings were not included.

**Table 1:** Clinical and laboratory characteristics of the study groups

Variable	PREGDM (*n* = 30), median [IQR]	GDM (*n* = 30), median [IQR]	Control (*n* = 30), median [IQR]	*p* value
Known diabetes duration, years	2.00 [1.00–3.00]	Not applicable	Not applicable	—
Age, years	28.50 [25.25-33.00]	32.50 [28.25-37.00]	26.00 [24.25-29.00]	0.005
Gestational age, weeks	8.00 [7.00–9.00]	27.00 [25.00–28.00]	18.00 [8.00–27.00]	< 0.001
BMI, kg/m²	29.20 [27.00-32.88]	30.85 [26.05–33.50]	29.00 [27.25-32.00]	0.809
TSH, mIU/L	2.54 [1.47–3.16]	1.61 [0.86–2.21]	2.92 [2.34–3.23]	< 0.001
Hemoglobin, g/dL	13.15 [12.33–13.75]	12.60 [12.12–13.35]	12.70 [12.00-13.30]	0.302
HbA1c, %	6.30 [5.78–6.93] (*n* = 28)	5.00 [4.80–5.45] (*n* = 30)	Not assessed	< 0.001
Neudesin, ng/mL	5.67 [4.72–6.21]	2.80 [2.70–3.10]	2.17 [1.77–2.89]	< 0.001

Continuous variables are presented as median [interquartile range]. Group comparisons were performed using the Kruskal-Wallis test, except HbA1c, which was compared between PREGDM and GDM using the Mann-Whitney U test. Diabetes duration was recorded only in the PREGDM group

*BMI *body mass index, *GDM *gestational diabetes mellitus, *PREGDM *pregestational type 2 diabetes mellitus, *TSH *thyroid-stimulating hormone

Multiple pregnancy, active infection, chronic inflammatory or autoimmune disease, malignancy, severe liver or kidney disease, serious systemic disease known before pregnancy, acute obstetric complications, refusal to participate, inability to provide blood samples, and incomplete clinical or laboratory data were exclusion criteria.

Demographic, obstetric, and clinical data were recorded from patient files and case report forms. These variables included age, height, body weight, body mass index (BMI), gravida, parity, number of abortions, number of living children, gestational age, blood group, comorbid conditions, medication use, family history, previous history of GDM, and diseases or symptoms developing during pregnancy.

Laboratory parameters assessed during routine clinical follow-up were recorded, including hemoglobin, white blood cell count, platelet count, HbA1c, fasting blood glucose, oral glucose tolerance test results, creatinine, thyroid-stimulating hormone (TSH), and urinalysis. These variables were evaluated as clinical and metabolic parameters that could be related to serum neudesin levels. In addition to routine clinical blood sampling, approximately 4 mL of venous blood was obtained for serum neudesin measurement. Samples were delivered to the central laboratory sample-processing unit and centrifuged at 4000 rpm for 10 min. The separated serum was aliquoted into labeled secondary tubes and stored at -20 °C until analysis. Following completion of sample collection, all serum samples were removed from storage on the evening before the assay day, allowed to thaw under the same conditions, and analyzed on the following day. Serum neudesin concentrations were measured in the Research Laboratory of the Department of Medical Biochemistry, Central Laboratory, Ankara Etlik City Hospital, using a commercially available human neudesin enzyme-linked immunosorbent assay kit (BT LAB, catalogue no. E4258Hu). Measurements were performed using a microplate reader, and absorbance was read at 450 nm. The assay was performed according to the manufacturer’s instructions using a sandwich ELISA principle. Microplate wells were pre-coated with antibodies specific for human neudesin. Neudesin in serum samples bound to the immobilized antibodies, followed by the addition of a biotinylated anti-human neudesin antibody and streptavidin-horseradish peroxidase. After incubation and washing steps, substrate solution was added, resulting in color development proportional to the neudesin concentration. The reaction was terminated with an acidic stop solution, and serum neudesin concentrations were calculated from the absorbance-concentration standard curve. According to the manufacturer, the assay sensitivity was 0.026 ng/mL, the measurement range was 0.05-24 ng/mL, and the reported intra-assay and inter-assay coefficients of variation were < 8% and < 10%, respectively. Samples were analyzed in single wells. All samples from the study groups were processed, stored, thawed, and analyzed under the same standardized laboratory conditions using the same assay kit and analytical platform, supporting the internal consistency of comparisons among study groups. Each participant contributed a single serum sample at the time of clinical evaluation; no longitudinal follow-up measurements were obtained.

### Ethics statement

The study protocol was approved by the Ethics Committee of Etlik City Hospital (AEŞH-BADEK1-2024-776). The study was conducted in accordance with the principles of the Declaration of Helsinki. Written informed consent was obtained from all participants before enrollment.

### Statistical analysis

Statistical analyses were performed using an appropriate statistical software package. The distribution of continuous variables was assessed using histograms, Q-Q plots, and the Shapiro-Wilk test. Continuous variables are presented as median [interquartile range], and categorical variables are presented as number (percentage).

Because of the sample size and distributional characteristics of several variables, three-group comparisons were performed using the Kruskal-Wallis test as an overall descriptive analysis. Pairwise comparisons were performed using the Mann-Whitney U test when an overall difference was found, and Bonferroni correction was applied for the primary pairwise comparisons. Pairwise neudesin differences were additionally summarized using Hodges-Lehmann median differences with 95% confidence intervals and rank-biserial correlation coefficients to support interpretation beyond p values. To address the clinically different sampling periods of the diabetic groups, prespecified gestational-timing-aware comparisons were used as the most clinically informative control contrasts: PREGDM was compared with controls sampled at 6–12 weeks, and GDM with controls sampled at 24–28 weeks. Categorical variables were compared using the chi-square test or Fisher’s exact test, as appropriate. Analyses that combined all control participants were interpreted cautiously because diagnostic group and gestational age remained partly associated by design. Associations between serum neudesin levels and selected clinical or metabolic variables were assessed using Spearman correlation analysis and were considered exploratory. HbA1c was not measured in the control group because it was not part of routine antenatal assessment in low-risk pregnancies and no additional research-specific laboratory testing was performed in healthy controls under the approved protocol. Therefore, the HbA1c-neudesin correlation was evaluated only in the diabetic groups. Correlation analyses were not interpreted as confirmatory, and individual correlations were interpreted cautiously in view of multiple comparisons.

As exploratory adjusted analyses, serum neudesin was natural-log transformed and evaluated using linear regression. The all-participant model included diagnostic group, gestational age, age, and BMI as covariates. A diabetic-only model compared PREGDM with GDM after adjustment for gestational age and age. Regression coefficients were exponentiated and reported as geometric mean ratios with 95% confidence intervals. Heteroscedasticity-robust standard errors were used. A sensitivity model additionally included TSH because TSH differed among groups. Treatment type was not included as a covariate because all women in the diabetic groups were sampled before pharmacological glucose-lowering treatment, leaving no between-participant variation in drug exposure. The all-participant regression model included gestational age as a linear covariate and was considered supportive and exploratory because non-linear gestational changes in neudesin could not be fully assessed in this sample. Therefore, gestational-window-restricted comparisons were additionally performed as clinically more informative sensitivity analyses. Given the modest sample size and clinical differences in sampling timing among groups, adjusted analyses were interpreted cautiously. A p-value of < 0.05 was considered statistically significant.

### Primary and secondary endpoints

The primary endpoint was the comparison of serum neudesin levels among pregnant women with PREGDM, pregnant women with GDM, and non-diabetic pregnant controls. Secondary exploratory endpoints included associations between serum neudesin and selected available clinical or metabolic variables, including HbA1c in the diabetic groups only. Additional exploratory endpoints included gestational-timing-aware comparisons and adjusted log-neudesin regression models.

## Results

A total of 90 pregnant women were included: 30 with PREGDM, 30 with GDM, and 30 non-diabetic controls. Age differed significantly among the groups (*p* = 0.005), and gestational age also differed significantly (*p* < 0.001). BMI was similar among the groups (*p* = 0.809). Baseline clinical and laboratory characteristics are summarized in Table 1.

At the time of serum sampling, all diabetic participants were managed with diet alone and had not received pharmacological glucose-lowering therapy. Known duration of type 2 diabetes in the PREGDM group was 2.00 [1.00–3.00] years (range, 1–4 years).

In laboratory analyses, TSH levels differed significantly among the groups (*p* < 0.001), whereas hemoglobin levels did not (*p* = 0.302). HbA1c was analyzed only in the diabetic groups and was higher in the PREGDM group than in the GDM group (6.30 [5.78–6.93]% vs. 5.00 [4.80–5.45]%; *p* < 0.001).

**Fig. 1:**
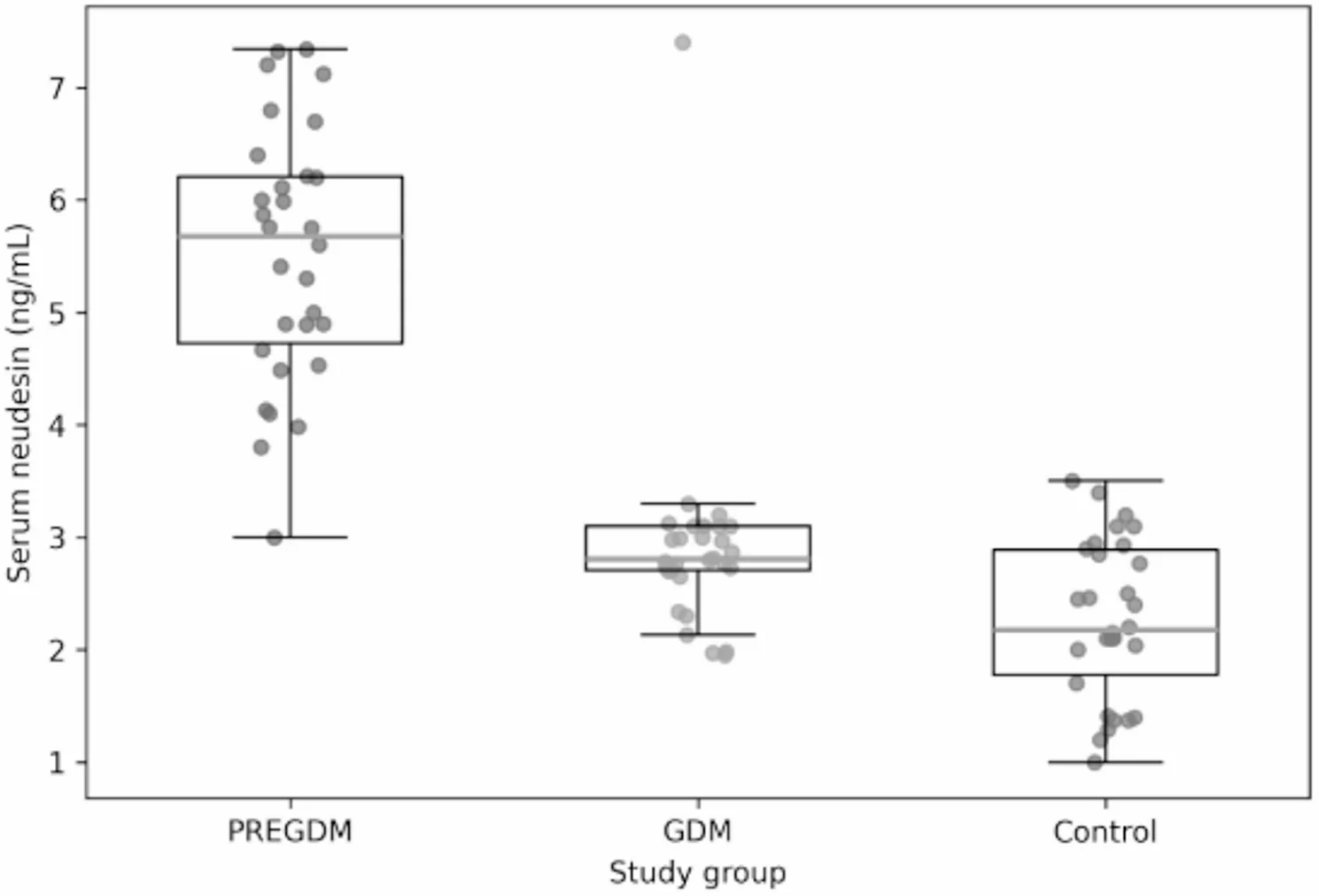
Distribution of serum neudesin concentrations by study group. Individual observations are overlaid on box plots; the central line indicates the median, box boundaries indicate the interquartile range, and whiskers extend to 1.5 times the interquartile range

Serum neudesin levels differed significantly among the three groups in the overall descriptive analysis (Kruskal-Wallis H = 57.87, *p* < 0.001; epsilon-squared = 0.64). Median neudesin was 5.67 [4.72–6.21] ng/mL in PREGDM, 2.80 [2.70–3.10] ng/mL in GDM, and 2.17 [1.77–2.89] ng/mL in controls. In Bonferroni-corrected pairwise comparisons using the overall control group, neudesin was higher in PREGDM than in GDM and controls (both adjusted *p* < 0.001). Although the GDM group also had higher neudesin than the overall control group (adjusted *p* = 0.017), this unstratified comparison required interpretation in view of the heterogeneous gestational timing of control sampling. Pairwise effect-size estimates are shown in Table 2. Figure 1 is a box-and-jitter plot displaying individual observations; box boundaries represent the interquartile range, the central line represents the median, and whiskers extend to 1.5 times the interquartile range.

**Table 2:** Overall pairwise comparison of serum neudesin levels

Comparison	Hodges–Lehmann difference, ng/mL	95% CI	Rank-biserial *r*	Bonferroni-adjusted *p*
PREGDM vs. GDM	2.77	2.15–3.22	0.91	< 0.001
PREGDM vs. Control	3.27	2.76–3.80	0.99	< 0.001
GDM vs. Control	0.58	0.17–0.88	0.42	0.017

Positive median differences indicate higher neudesin levels in the first group of the comparison

*CI *confidence interval

Because gestational age differed among groups, gestational-timing-aware exploratory comparisons were performed. PREGDM was compared with early-pregnancy controls sampled at 6–12 weeks (*n* = 15), and GDM was compared with controls sampled at 24–28 weeks (*n* = 15). PREGDM remained markedly higher than early-pregnancy controls (5.67 [4.72–6.21] vs. 1.70 [1.37–2.15] ng/mL; Hodges-Lehmann difference 3.70 ng/mL; 95% CI, 3.15–4.35; *p* < 0.001). In contrast, GDM did not differ from controls sampled at 24–28 weeks (2.80 [2.70–3.10] vs. 2.85 [2.28–3.03] ng/mL; Hodges-Lehmann difference 0.04 ng/mL; 95% CI, -0.20 to 0.40; *p* = 0.791). In a diabetic-only log-neudesin model adjusted for gestational age and age, the PREGDM-GDM contrast was attenuated and was not statistically significant (GMR 1.31; 95% CI, 0.61–2.85; *p* = 0.490). Because PREGDM and GDM were sampled at systematically different gestational ages, this adjusted diabetic-only estimate should be interpreted cautiously because of possible collinearity and overadjustment. These timing-aware analyses are summarized in Table 3. In the pooled diabetic sample, HbA1c was positively correlated with neudesin (rho = 0.531, *p* < 0.001). However, correlations within PREGDM (rho=-0.203, *p* = 0.300) and within GDM (rho = 0.064, *p* = 0.736) were not significant. Therefore, the pooled association is interpreted as predominantly a between-group pattern driven by the higher HbA1c and neudesin values in PREGDM relative to GDM, rather than as evidence of a consistent within-group HbA1c-neudesin relationship. In exploratory multivariable log-neudesin regression adjusted for gestational age, age, and BMI across all participants, PREGDM remained associated with higher neudesin than controls (GMR 3.11; 95% CI, 2.57–3.76; *p* < 0.001), whereas the GDM-control contrast was not statistically significant (GMR 1.12; 95% CI, 0.96–1.30; *p* = 0.149). Gestational age was also positively associated with neudesin in this model (GMR per week 1.02; 95% CI, 1.01–1.03; *p* < 0.001). The adjusted PREGDM-control estimate is supportive but does not fully remove the design-related concern about gestational-age confounding; it should be interpreted together with the early-pregnancy comparison rather than as evidence of a gestational-age-independent trajectory across pregnancy. Full regression results are shown in Table 4. A sensitivity model that additionally included TSH yielded similar findings (PREGDM vs. control GMR 3.10; 95% CI, 2.56–3.76; *p* < 0.001; GDM vs. control GMR 1.11; 95% CI, 0.96–1.29; *p* = 0.168).

**Table 3:** Gestational-timing-aware exploratory comparisons of serum neudesin levels

Exploratory analysis	Groups compared	Effect estimate	95% CI	*p* value
PREGDM vs. early-pregnancy controls	PREGDM (*n* = 30): 5.67 [4.72–6.21] vs. controls 6–12 weeks (*n* = 15): 1.70 [1.37–2.15] ng/mL	Hodges-Lehmann difference 3.70 ng/mL	3.15–4.35	< 0.001
GDM vs. 24-28-week controls	GDM (*n* = 30): 2.80 [2.70–3.10] vs. controls 24–28 weeks (*n* = 15): 2.85 [2.28–3.03] ng/mL	Hodges-Lehmann difference 0.04 ng/mL	-0.20-0.40	0.791
PREGDM vs. GDM adjusted diabetic-only model	PREGDM vs. GDM among diabetic participants (*n* = 60)	GMR 1.31	0.61–2.85	0.490

Values are presented as median [interquartile range]. Positive Hodges-Lehmann differences indicate higher neudesin levels in the first group. The diabetic-only model used natural-log transformed neudesin and adjusted for gestational age and age; GDM was the reference group

*CI *confidence interval, *GMR *geometric mean ratio

**Table 4:** Exploratory multivariable log-neudesin regression in all participants

Variable	Geometric mean ratio	95% CI	*p* value
GDM vs. control	1.12	0.96–1.30	0.149
PREGDM vs. control	3.11	2.57–3.76	< 0.001
Gestational age, per week	1.02	1.01–1.03	< 0.001
Age, per year	0.99	0.98-1.00	0.125
BMI, per kg/m²	1.00	1.00-1.01	0.477

Dependent variable was natural-log transformed serum neudesin. The model included diagnostic group, gestational age, age, and BMI, and used heteroscedasticity-robust standard errors. Control group was the reference category. Model R²=0.745

*CI *confidence interval, *GDM *gestational diabetes mellitus, *PREGDM *pregestational type 2 diabetes mellitus

## Discussion

This exploratory cross-sectional controlled study compared serum neudesin levels among pregnant women with GDM, pregnant women with PREGDM, and non-diabetic pregnant controls. The most consistent signal within this dataset was observed for PREGDM. This difference was large in non-parametric analysis, remained evident in the early-pregnancy control comparison, and persisted in the all-participant log-neudesin regression model adjusted for gestational age, age, and BMI. By contrast, the GDM-control difference was no longer evident when GDM was compared specifically with controls sampled at 24–28 weeks and was not significant in the adjusted all-participant model. Therefore, the GDM finding should be considered exploratory and timing-sensitive rather than confirmatory biomarker evidence.

Gestational timing remains an important consideration in interpreting this study. Sampling occurred predominantly in early pregnancy in the PREGDM group and around 24–28 weeks in the GDM group. To provide clinically appropriate comparisons, the control group was intentionally recruited in two prespecified gestational-age strata corresponding to these sampling periods, with 15 controls sampled at 6–12 weeks and 15 controls at 24–28 weeks. Nevertheless, when all participants were analyzed together, gestational age remained independently associated with neudesin (GMR per week 1.02; 95% CI, 1.01–1.03; *p* < 0.001) and was partly collinear with diagnostic group by design. The timing-stratified analyses and adjusted regression therefore provide complementary information, but the cross-sectional design cannot completely separate diagnostic-group effects from gestational timing. In particular, adjustment for gestational age in the diabetic-only PREGDM–GDM model may attenuate part of the clinically inherent timing difference between these groups. Furthermore, the linear gestational-age term may not fully capture non-linear biological changes in neudesin across pregnancy.

Diabetes in pregnancy is clinically important because of its maternal and fetal consequences. Current guidelines and reviews emphasize that both GDM and pre-existing diabetes are associated with adverse pregnancy outcomes, although the timing of metabolic exposure differs substantially [1, 2, 16–18]. In GDM, insulin resistance develops against the background of pregnancy-related metabolic adaptation. In pregestational diabetes, hyperglycemia and insulin resistance are already present before conception, which may lead to a longer and more pronounced metabolic burden [1, 16–18]. This distinction may partly explain the higher neudesin levels observed in the PREGDM group.

Neudesin has been linked to energy metabolism, adipose tissue function, obesity, glucose metabolism, and insulin sensitivity [9–12]. In adults with type 2 diabetes, higher serum neudesin levels have been reported and interpreted in relation to insulin resistance and metabolic dysfunction [12]. Pediatric and adolescent studies, however, suggest that neudesin may behave differently across age groups, obesity phenotypes, and metabolic states [10, 11]. Thus, neudesin should not be viewed as a simple one-directional marker of adiposity. The present findings are more consistent with the view that neudesin may reflect broader metabolic disturbance, particularly when chronic diabetes is present before pregnancy.

The higher HbA1c level in the PREGDM group is consistent with a greater chronic glycemic burden. Although HbA1c and neudesin were positively correlated in the pooled diabetic sample, this relationship was not present within the PREGDM or GDM groups. The pooled correlation should therefore be interpreted primarily as a between-group association, reflecting separation of the PREGDM and GDM groups on both variables, rather than as evidence of a consistent within-group biological relationship between HbA1c and neudesin. The absence of HbA1c measurements in controls further precludes an across-group assessment of this relationship. Accordingly, these correlation findings are hypothesis-generating and should not be interpreted as confirmatory evidence of an independent HbA1c-neudesin association.

Previous studies evaluating neudesin in GDM have reported heterogeneous findings [13–15]. Eren et al. found higher maternal and umbilical cord neudesin levels in pregnancies with GDM [13]. Esmail et al. reported higher serum neudesin in GDM and positive associations with glycemic and insulin resistance indices [14]. Moussa et al. similarly reported increased maternal and cord blood neudesin in GDM and described correlations with BMI, insulin resistance measures, and neonatal parameters [15]. In the present dataset, the overall GDM-control difference became non-significant when the comparison was restricted to controls sampled at 24–28 weeks. Differences among studies may partly reflect variation in study population, gestational timing, sample handling, assay kits, calibration ranges, analytical sensitivity, and reporting units; therefore, absolute neudesin concentrations should not be compared across studies as if a common reference range had been established. Published studies also do not consistently provide directly comparable assay-performance details, which further limits cross-study calibration. Beyond neudesin, recent literature emphasizes the complex pathophysiology of GDM and the need to refine diagnostic and prognostic markers beyond conventional glycemic criteria [19, 20]. Updated adipokine-focused evidence on chemerin and first-trimester biomarker panels further supports the relevance of adipose-tissue-derived mediators in GDM risk stratification [21, 22]. Mechanistic reviews and studies of visceral or brown-fat-related adipokines, including omentin, vaspin, visfatin, and neuregulin 4, also highlight the broader metabolic context in which neudesin should be interpreted [23–25].

The present study adds to the literature by including a PREGDM group, which has been less frequently examined than GDM in previous neudesin studies. This pattern may be compatible with differences in chronic metabolic exposure and glycemic burden; however, diabetes duration was relatively short in the PREGDM group (median 2.00 [1.00–3.00] years; range, 1–4 years), and several pre-pregnancy metabolic factors were not available for direct assessment. The diabetic-only adjusted model comparing PREGDM with GDM was attenuated after adjustment for gestational age and age and should be interpreted cautiously because of the clinically different sampling times and potential collinearity. Restriction to untreated, diet-managed diabetic participants reduced concern that pharmacological treatment accounted for the observed neudesin differences. Because diabetes duration was defined only in the PREGDM group and the sample was modest, it was not included as a covariate; residual confounding by chronic metabolic exposure cannot be excluded.

BMI did not differ significantly among the three groups, and BMI was not independently associated with log-neudesin in the adjusted model. Therefore, the observed differences in neudesin levels cannot be explained only by maternal BMI. However, BMI is an imperfect measure of adipose tissue function and does not capture body fat distribution, gestational weight gain, insulin levels, or HOMA-IR. The absence of insulin and HOMA-IR measurements is particularly important because the central biological premise of the study concerns insulin resistance; this mechanism could not be directly tested in the present dataset. The significant difference in TSH among groups should also be acknowledged as a possible confounding factor, although a sensitivity model including TSH did not materially change the group estimates. Taken together, neudesin seems more likely to be a complementary metabolic marker than a standalone diagnostic marker.

The main strength of this study is the inclusion of a PREGDM group in addition to GDM and non-diabetic controls. Most studies on neudesin in pregnancy have focused on GDM, while pregestational diabetes has received less attention. Another strength is the controlled cross-sectional design and measurement of neudesin in the same clinical setting.

This study has limitations. It was conducted at a single center with a modest sample size, and age differed among groups. The study was not designed as a fully matched biomarker-validation cohort, and no formal a priori sample-size calculation was performed for a prespecified neudesin effect. For interpretive context, the GDM versus 24-28-week control comparison (*n* = 30 vs. *n* = 15) would have approximately 80% power to detect only a large standardized difference (Cohen’s d approximately 0.91) at a two-sided alpha of 0.05; therefore, small-to-moderate GDM-control differences cannot be excluded. PREGDM was assessed predominantly in early pregnancy, whereas GDM was sampled at diagnosis, usually at 24–28 weeks. Although controls were recruited in corresponding strata, gestational age remained partly associated with diagnostic group by design; therefore, timing-stratified comparisons are the most clinically appropriate contrasts and adjusted models are supportive exploratory analyses. The size of each control stratum limited precision and did not permit conclusions about a gestational neudesin trajectory. HbA1c was not measured in controls because it was not part of routine care for low-risk pregnancies, and no additional research-specific testing was performed under the approved protocol. Insulin levels and HOMA-IR were unavailable; therefore, insulin resistance could not be directly assessed. Serum samples were measured once. Although all samples were analyzed using the same assay kit and analytical platform, detailed retrospective documentation of plate-level sample allocation and run sequence was not available; therefore, a residual contribution of unrecorded plate- or run-level analytical variation cannot be completely excluded. Finally, the cross-sectional design does not permit causal inference. To reduce treatment-related confounding, the PREGDM group was restricted to women with no current or prior exposure to pharmacological glucose-lowering agents, and GDM samples were obtained before pharmacological treatment was initiated. Known diabetes duration was recorded in PREGDM (median 2.00 [1.00–3.00] years; range, 1–4 years), but could not be included in cross-group adjusted models because it was defined only in the PREGDM group. Residual confounding from cumulative metabolic exposure or other unmeasured pre-pregnancy factors remains possible.

Although gestational age was included as a linear covariate in the all-participant model, residual confounding due to non-linear changes in neudesin across pregnancy cannot be fully excluded. Accordingly, the gestational-window-matched comparisons were considered the more clinically informative analyses.

## Conclusion

Serum neudesin levels were markedly higher in pregnant women with pregestational type 2 diabetes. The most consistent signal within this dataset was observed for PREGDM, particularly in comparison with early-pregnancy controls and in the all-participant adjusted model. By contrast, the apparent GDM-control difference in the overall comparison was not maintained when GDM was compared with controls sampled at 24–28 weeks and was not significant in the adjusted all-participant model. Therefore, the present data do not support a gestational-age-robust elevation of neudesin in GDM. Overall, neudesin may reflect metabolic disturbance in diabetic pregnancies, particularly when diabetes predates conception, but larger prospective studies with gestational-age-matched controls, insulin resistance measurements, duplicate sample assays, and adjusted analyses are needed before neudesin can be proposed as a reliable diagnostic biomarker for diabetic pregnancy.

## Data Availability

The datasets generated and/or analysed during the current study are not publicly available due to privacy and ethical restrictions, as they contain participant-level clinical and laboratory data. However, anonymized data may be made available from the corresponding author on reasonable request and with appropriate institutional approval.
